# Radar Composite Reflectivity Reconstruction Based on FY-4A Using Deep Learning

**DOI:** 10.3390/s23010081

**Published:** 2022-12-22

**Authors:** Ling Yang, Qian Zhao, Yunheng Xue, Fenglin Sun, Jun Li, Xiaoqiong Zhen, Tujin Lu

**Affiliations:** 1 College of Electronic Engineering, Chengdu University of Information Technology, Chengdu 610225, China; 2 CMA Key Laboratory of Atmospheric Sounding, Chengdu 610225, China; 3 Key Laboratory of Radiometric Calibration and Validation for Environmental Satellites and Innovation Center for FengYun Meteorological Satellite (FYSIC), National Satellite Meteorological Center (National Center for Space Weather), China Meteorological Administration, Beijing 100081, China; 4 Hainan Meteorological Observation Center, Haikou 570203, China

**Keywords:** FY-4A geostationary meteorological satellite, deep learning, radar composite reflectivity

## Abstract

Weather radars are commonly used to track the development of convective storms due to their high resolution and accuracy. However, the coverage of existing weather radar is very limited, especially in mountainous and ocean areas. Geostationary meteorological satellites can provide near global coverage and near real-time observations, which can compensate for the lack of radar observations. In this paper, a deep learning method was used to estimate the radar composite reflectivity from observations of China’s new-generation geostationary meteorological satellite FY-4A and topographic data. The derived radar reflectivity products from satellite observations can be used over regions without radar coverage. In general, the deep learning model can reproduce the overall position, shape, and intensity of the radar echoes. In addition, evaluation of the reconstruction radar observations indicates that a modified model based on the attention mechanism (Attention U-Net model) has better performance than the traditional U-Net model in terms of all statistics such as the probability of detection (POD), critical success index (CSI), and root-mean-square error (RMSE), and the modified model has stronger capability on reconstructing details and strong echoes.

## 1. Introduction

Heavy rain, hail, lightning, and other strong convective weather events usually have the characteristics of strong intensity, rapid development, and wide distribution, thus causing high impacts on people’s lives and economic development [1,2,3]. Warning and responding to severe convective weather events require accurate and timely observations with high spatial and temporal resolutions. Weather radar detects strong convective weather characteristics using the echo signals from the emitted pulse waves reflected back by cloud and rain particles [4,5,6]. With high accuracy and spatio-temporal resolution, weather radar has always been one of the most powerful tools for monitoring severe convective weather and is widely used in operational systems. Radar reflectivity reflects the size and density distribution of precipitation particles, and thus is often used to represent the intensity of weather targets. In general, the larger the reflectivity intensity, the higher probability of strong convective weather. Studies have shown that radar reflectivity greater than 35 dBZ (dBZ: a physical quantity that represents the intensity of radar echoes) tends to indicate the occurrence of severe convective weather [7]. The radar echo images generated based on the radar reflectivity factor provide a more intuitive indication of the occurrence and development of convective systems [8]. Furthermore, the radar reflectivity factor is widely used in numerical weather prediction (NWP) models and data assimilation systems to further improve the forecasts of clouds and precipitation [9,10].

However, complicated topography (especially hills and mountains) often brings uncertainties into radar measurements [11]. Moreover, radars are mainly deployed in densely populated areas, for example, radars are deployed over east China, while southwest China still lacks radar observations due to the complex topography and large uninhabited areas. Therefore, it is more difficult to monitor and forecast severe convective weather in southwest China.

In contrast, an advanced imager onboard the geostationary (GEO) meteorological satellite, as a space-based remote sensing instrument, is not restricted by complex topography and natural conditions [12]. Observations from the visible (VIS) and infrared (IR) bands involve information on the development of cloud tops. Generally, the stronger the convections that develop, the higher their cloud top will be. Moreover, the thicker the cloud, the stronger the precipitation and the lower the brightness temperature (BT) observed by the satellite IR bands [13]. Therefore, GEO meteorological satellites can monitor the development of convective cloud systems [14,15]. In particular, the new generation of GEO satellites has much improved spatio-temporal resolution and enhanced capabilities for monitoring the rapid development of severe convective weather processes when compared with the last generation of GEO weather satellites. Launched in 2016, Fengyun-4A (FY-4A) is the first satellite of the Chinese new-generation GEO meteorological satellite (FengYun-4 series). It carries a radiation imager named the Advanced Geosynchronous Radiation Imager (AGRI), the performance of which has significantly improved compared with the previous one onboard Fengyun 2 satellites [16]. The AGRI has been upgraded from 5 to 14 spectral bands, with the spatial resolution ranging from 0.5 km (for VIS bands) to 4 km (for IR bands). The observation time for full-disk scan mode is reduced from 0.5 h to 15 min, and the Chinese domain can be scanned every 5 min [17] which is close to the 6 min scan of weather radars. Therefore, it is physically feasible to reconstruct radar reflectivity using geostationary satellite imager observations with a much wider geographical coverage, which can provide observations without radar and fulfill the missing data in the blind areas of radar screening. It can also be blended with real radar observations in areas within radar coverage to provide higher frequency observations for monitoring rapidly developing strong convective systems.

Satellite IR band observations mainly contain cloud top radiation information and the radiances holds limited information for cloud structure and precipitating since the IR bands have limited capability to penetrate the clouds, this limits the physical retrieval of clouds’ optical, microphysical, and precipitation characteristics. VIS bands can provide more insight cloud information but are limited to the observations during daytime. Studies have shown that the maximum cloud optical thickness (COT) generated by physical-based retrieval algorithms is about 160, which roughly corresponds to only 20–25 dBZ of the radar reflectivity factor [18]. In contrast, the convolutional neural network (CNN) machine learning (ML) technique has the ability to capture the image gradient features and is able to reconstruct radar echoes exceeding 50 dBZ [19]. Therefore, the ML technique is able to compensate for the limitation of physical retrieval algorithms to some extent. In recent years, deep learning (DL) technology has been widely used in meteorological studies. For example, a CNN was used to classify and predict strong convective weather and precipitation with satellite and radar-based observations [20,21,22]. A generative adversarial network (GAN) was used for the extrapolation of satellite cloud and radar echo maps [23,24] and some studies have been conducted on reconstructing the high-resolution data with ML algorithms, etc. [25]. Compared with traditional ML algorithms, DL algorithms contain deep CNNs with more hidden layers [26]. Therefore, they have better high-dimensional nonlinear modeling performance. Deep neural networks can not only learn spatial information but also the time-varying information contained in data with time-series characteristics, and further, make predictions based on the captured features.

Recently, several studies have been presented using DL techniques to reconstruct radar composite reflectivity (CREF) [19,27,28]. This study is a further exploration based on the previous works, a DL technology is developed to reconstruct radar CREF from observations of China’s new-generation geostationary meteorological satellite FY-4A. This model is a regional model with a focus on the weather conditions in southwest China, as previous studies indicate that strong convections have distinct local characteristics. To consider the complex surface of southwestern China, the topography data are also added to the model. Moreover, a new DL model based on the attention mechanism is introduced to improve the reconstruction ability and a comparison with the traditional U-Net model (which is the convolutional network architecture for fast and precise segmentation of image) is also conducted. The reconstructed CREF maps from the GEO satellite imager observations can be applied to monitoring large-scale weather systems and the early warning of severe local storms, and provide more complete information on rapidly developing severe convective weather systems as well as meteorological disasters.

Section 2 is the introduction of data used in this paper. Section 3 mainly focuses on the data preprocessing, the structure of two DL models, and model evaluation methods. The independent validation results and typical case study, along with real application demonstration are presented in Section 4. A summary and conclusions are shown in Section 5.

## 2. Data

### 2.1. Satellite Observations

FY-4A is the first of the new generation of Chinese geostationary meteorological satellites (FengYun-4 series) and was successfully launched on 11 December 2016, positioned around 104.7° E [16]. The FY-4A carries an Advanced Geosynchronous Radiation Imager (AGRI). The AGRI was designed with 14 spectral bands, including VIS bands (with a central wavelength from 0.47 μm to 0.65 μm), near-infrared (NIR) bands (with central wavelengths from 0.825 μm to 2.225 μm), and IR bands (with central wavelengths from 3.725 μm to 13.5 μm), which can perform a 5 min scan over Chinese regions and a 15 min scan in full disk [17]. The spatial resolution ranges from 0.5 km (VIS band) to 4 km (IR band) at the nadir. The VIS and NIR bands contain more information about clouds, but it is only available during the daytime. In contrast, the IR bands can provide observations during both daytime and nighttime. Therefore, two models were established to fully use the cloud information contained in all available bands. One model uses the VIS/NIR and IR bands as input (termed the VIS+IR model) for the daytime, and the other one only uses IR bands as input (termed the IR-only model) for the whole-day reconstructions. Table 1 shows the input parameters and corresponding physical meanings of the VIS+IR model and IR-only model, respectively.

The input imager spectral bands in the model are selected according to the physical interpretation of radar reflectivity–convection–satellite observation relationships, mainly focusing on bands that are most sensitive to clouds and hydrometeors, and the study of Sun et al. [28] was also referred to. For example, the VIS band at 0.65 μm is a weak absorption band sensitive to COT and cloud phase, especially for strong convective clouds. The 1.61 μm band in the NIR spectral region shows high sensitivity to ice-phase clouds and cloud-effective particle radius with a strong absorption effect. Moreover, clouds with different phases have different absorption characteristics in the 8.6, 10.8, and 12.0 μm bands. While the brightness temperature difference (BTD) of 12.3–10.8 μm is less than that of μm for cloud water particles, the opposite situation is obtained for cloud ice particles. Based on those situations, the VIS+IR model chooses VIS band of 0.65 μm, NIR band of 1.61 μm, and IR bands of 10.8 μm, 12.3 μm along with BTDs of 10.8–6.2 μm and 12.3 + 8.6 − 2 × 10.8 μm combination as model input. For the IR-only model, only IR bands are used as input to produce unified day/night results. Note that the VIS and NIR band observations have been modified by multiplying the sec(θ), where θ is the solar zenith angle (θ < 65${}^{\circ }$). Some studies have found that the height of the ground surface may have some influence on the effect of the model [27], so the digital elevation model (DEM) data are also included as an input parameter in this study to represent the topographic information.

### 2.2. Radars

The radar data used in this study are obtained from the China New Generation Weather Radar Network (CINRAD) deployed by the China Meteorological Administration (CMA). CINRAD contains 123 S-band and 94 C-band Doppler weather radars. S-band radars are mainly deployed in the eastern and coastal regions, while C-band radars are deployed primarily in the northwest and northeast regions. These radars provide accurate monitoring and forecasting of weather disasters such as typhoons, thunderstorms, and hail [29]. Since this study aims to build a regional radar reflectivity retrieval model, especially for the southwest of China, the CREF data in southwest China range from 25° N to 35° N latitude, and 100° E to 110° E longitude was finally chosen as the label data. This region was selected because the radar coverage is larger than most other areas in western China, thus providing sufficient radar observations for model training. CREF is the maximum base reflectivity factor that can be found in a given vertical column in the radar umbrella [28]. CREF can visually reflect the intensity structure and variation of a strong convective system. The radar CREF data have a temporal resolution of 6 min and a spatial resolution of 0.01° with the maximum value of 67 dBZ in the labeled dataset.

### 2.3. GPM Precipitation Data

Because precipitation and radar echoes have a good relationship, to test the effectiveness of radar echoes reconstructed by the DL model in areas without radar coverage, the Global Precipitation Measurement (GPM) precipitation dataset is selected to test the reconstruction result of the DL model. The GPM is the next generation of the Global Satellite Precipitation Measurement Program following the Tropical Rainfall Measurement Mission (TRMM) precipitation program, with a constellation of 10 satellites launched on 28 February 2014 [30,31]. The GPM carries an advanced dual-frequency rain gauge radar and passive microwave sensors, which can detect solid and slight precipitation more accurately [32]. Attributed to the fusion of multiple satellites and rain gauge data, the accuracy of GPM precipitation data is relatively high among other satellite precipitation products [30]. The precipitation dataset used in this study is the latest generation of Integrated Multi-Satellite Retrievals for GPM (IMERG). Based on different algorithm processing, GPM IMERG provides three types of precipitation data, which are early, late, and final runs with latencies of 4 h, 12–24 h, and 3.5 months, respectively. The final run uses a month-to-month adjustment to combine the multi-satellite data with Global Precipitation Climate Center (GPCC) gauge. In this study, the final run dataset with a spatial resolution of 0.1° × 0.1° and a temporal resolution of 30 min was used [33].

## 3. Method

**Figure 1:**
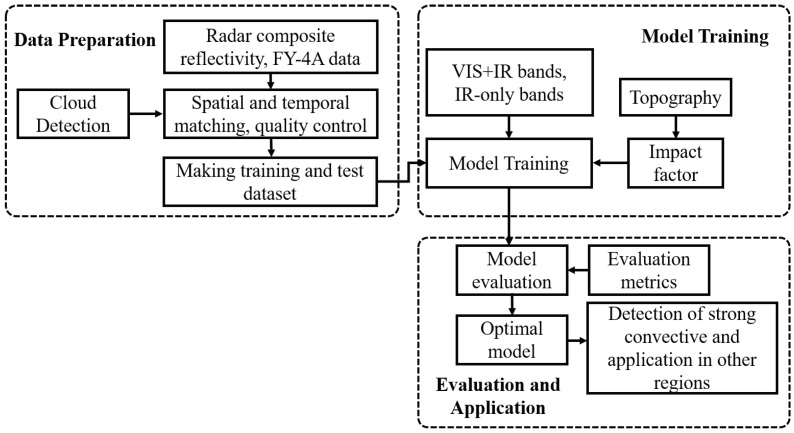
Workflow of CREF reconstruction algorithm based on the DL technique.

Figure 1 illustrates the workflow of this study. It consists of three modules: data selecting and preprocessing, model training, and model evaluation based on independent datasets.

### 3.1. Data Preprocessing

**Figure 2:**
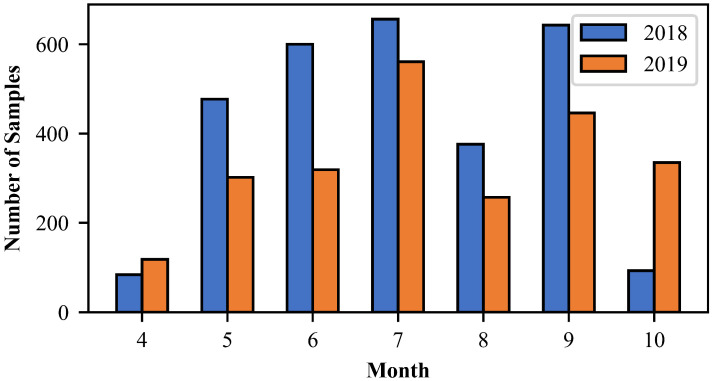
Number of samples for model training and validation in each month of 2018–2019.

The FY-4A AGRI observations and radar CREF data are selected from the warm seasons from April to October 2018 and 2019 when there were abundant convective activities. FY-4A AGRI VIS/IR band observations and cloud mask (CLM) products are used in this study. The spatial resolution of AGRI is 4 km and the time resolution is 5 min. While the radar data have a higher spatial resolution of 0.01° × 0.01°, they are interpolated to the 0.04° × 0.04° grid resolution to match the AGRI observations. The time difference between matched satellite and radar observations is less than 3 min. Since there are too many clear sky pixels from one satellite image, which are distractions for the CNN model, the operational CLM product from AGRI is then adopted to exclude these clear pixels. The AGRI CLM product is classified into four categories [34]: 1—cloudy, 2—probably cloudy, 3—clear sky, and 4—probably clear sky. Only those pixels in the first category are treated as cloudy pixels in this study, and all other pixels are considered non-cloudy sky cases that will be discarded. After the spatiotemporal collocation and the exclusion of clear-sky pixels, 5267 samples containing convective events are finally obtained. The matched datasets are randomly split into 80% (about 4223 samples) as the training dataset and 20% (about 1044 samples) as the independent validation dataset. Figure 2 shows the distribution of samples in each month. Finally, the maximum and minimum normalization is applied, which scales the input data to the range of 0 to 1 to accelerate the model convergence.

### 3.2. DL Network Structure

#### 3.2.1. U-Net

**Figure 3:**
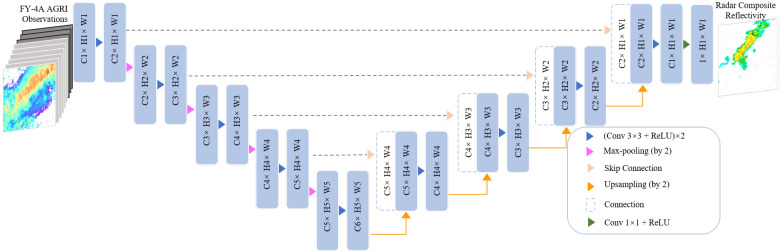
Strcucture of U-Net.

The U-Net network is originally applied in the field of semantic segmentation (the process of assigning a class label to each pixel in an image) [35]. Based on the framework of the fully convolutional network, U-Net uses an encoder–decoder structure in order to better capture the multi-scale contextual information of the images. The network structure with some modifications is widely used for regression prediction in the field of meteorology. As illustrated in Figure 3, the U-Net model consists of two parts: an encoder structure (left side) and a decoder structure (right side). The encoder structure is mainly used for feature extraction and includes several convolution blocks. Each convolution block contains two 3 × 3 convolution layers, an activation function named the rectified linear unit (ReLU), and a max-pooling layer with a stride of 2 for down-sampling. At each down-sampling step, the number of feature channels is doubled. The decoder structure contains key steps of up-sampling which halve the number of feature channels and skip connection. The encoder and decoder structures are connected by the skip link structure to combine low-dimensional feature maps with high-dimensional feature maps. At the last layer, a 1 × 1 convolution layer is used to map the feature vectors to the assigned regression map. The sizes of input data are 251 × 251 × 9 for the VIS+IR model and 251 × 251 × 6 for the IR-only model.

#### 3.2.2. Attention U-Net

**Figure 4:**
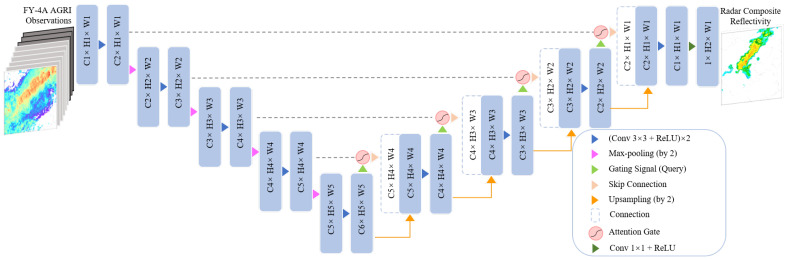
Strcucture of Attention U-Net.

Attention U-Net is a transformation of U-Net which introduces an attention mechanism to process encoder and decoder feature maps. As shown in Figure 4, the U-Net model is the main architecture, and the attention gates denoted by the red circles are integrated to automatically adjust the feature weight in different locations before skipping the connection of the encoder and decoder [36]. Compared to the U-Net networks, Attention U-Net can focus on the region of interest by putting more weight on features that are passed through the skip connections. Moreover, the Attention U-Net does not introduce significant additional computation. Oktay et al. [37] showed that after combining the attention mechanism with U-Net, the model could suppress feature activations in irrelevant regions during the learning process and can finally achieve better performance than before.

### 3.3. Model Training and Testing

The model inputs for the IR-only model and VIS+IR model are 6 bands and 9 bands, respectively. The model output is the composite radar reflectivity map. Both satellite data and the composite radar reflectivity have a size of 251 pixels in width and 251 pixels in height. In this study, the Attention U-Net model (marked as AU model) and the U-Net model (marked as U model) are built for the IR-only model and VIS+IR model, respectively. The models are built with Pytorch, and the optimizer is Adam, which can make the loss function reach the optimal value as soon as possible. Moreover, a batch input method is used to accelerate the training speed, and the batch size is set to 4. In the training model, the learning rate is initially set to 0.0005. During the training process, the learning rate is adjusted by the “warm-up” adjustment strategy [38,39] to avoid the oscillation of the loss function curve due to the high initial learning rate setting. The training is set to 300 epochs, and it will stop when the loss function ceases to fall ten times in succession. The loss function is designed as the sum of the root-mean-square error (RMSE) and the absolute error (MAE). In addition, RMSE, MAE, and explained variance (R2) are also calculated to evaluate the performance of the model. Lower RMSE and MAE indicate a smaller difference between actual radar CREF and reconstructed values, which means the models have a better performance. Similarly, the closer R2 is to 1, the better the model is. RMSE, MAE, and R2 are expressed as Equations (1)–(3), respectively. In these equations, $y_{i}$ and $y_{pred}$ are the actual CREF and reconstructed CREF from satellite observations. $\bar{y_{i}}$ is the average of the actual radar observations.

(1) $$\mathrm{RMSE}=\sqrt{\frac{\sum_{i=1}^{N}{\left(y_{i}-y_{pred,i}\right)}^{2}}{N}}$$

(2) $$\mathrm{MAE}=\frac{\sum_{i=1}^{N}\left(\left|y_{i}-y_{pred,i}\right|\right)}{N}$$

(3) $$R2=1-\frac{\sum_{i=1}^{N}{\left(y_{i}-y_{pred,i}\right)}^{2}}{\sum_{i=1}^{N}\left(y_{i}-\bar{y_{i}}\right)}$$

Different radar echo intensities are usually given different attention in meteorology. Therefore, it is necessary to evaluate the model at different radar intensities. Classification metrics including the probability of detection (POD), false alarm ratio (FAR), critical success index (CSI), and Heidke skill score (HSS) are utilized here to evaluate the reconstruction performance of the model at different thresholds of CREF. Higher POD, CSI, and HSS values and smaller FAR values indicate better model performance. The definition of used classification scores is defined in Table 2 and Equations (4)–(7).

(4) $$\mathrm{POD}=\frac{\mathrm{TP}}{\mathrm{TP}+\mathrm{FN}}$$

(5) $$\mathrm{FAR}=\frac{\mathrm{FP}}{\mathrm{FP}+\mathrm{TP}}$$

(6) $$\mathrm{CSI}=\frac{\mathrm{TP}}{\mathrm{TP}+\mathrm{FP}+\mathrm{FN}}$$

(7) $$\mathrm{HSS}=\frac{2\times (\mathrm{TP}\times \mathrm{TN}-\mathrm{FN}\times \mathrm{FP})}{\mathrm{FN}\times 2+\mathrm{FP}\times 2+2\times \mathrm{TP}\times \mathrm{TN}+(\mathrm{FN}+\mathrm{FP})\times (\mathrm{TP}+\mathrm{TN})}$$

## 4. Results

### 4.1. Statistical Results

**Figure 5:**
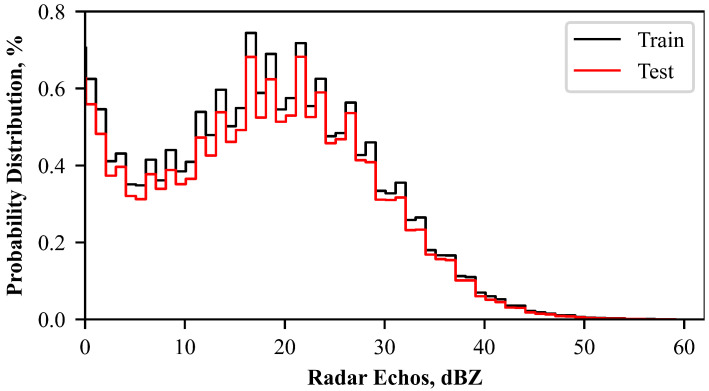
Probability distribution of CREF in the training (black line) and test (red line) datasets, respectively. Note that 0 dBZ is not included.

Figure 5 shows the probability distribution of nonzero radar reflectivity in all samples. The results show that the distribution of training and test samples is relatively consistent, which means the test set can represent the features of the training set. Note that the CREF is mainly concentrated around 0–30 dBZ, with a small proportion of strong radar echoes exceeding 35 dBZ.

**Figure 6:**
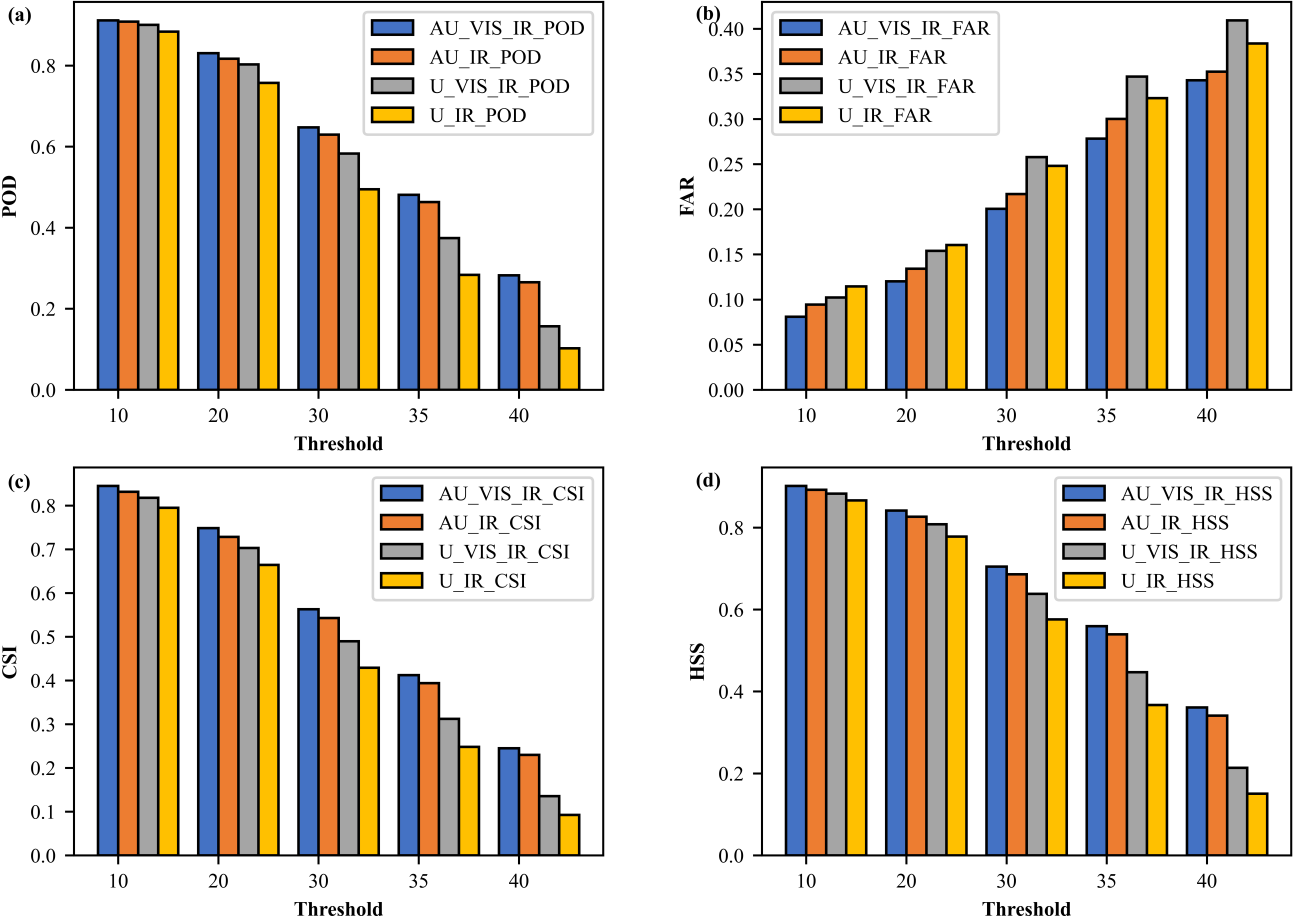
Classification metrics of four models in different radar echo thresholds. (**a**) POD; (**b**) FAR; (**c**) CSI; (**d**) HSS. AU_VIS_IR is the Attention U-Net VIS+IR model, AU_IR is the Attention U-Net IR-only model, U_VIS_IR is the U-Net VIS+IR model, and U_IR is the U-Net IR-only model.

In this study, four models are established, namely, the Attention U-Net VIS+IR model, Attention U-Net IR-only model, U-Net VIS+IR model, and U-Net IR-only model. To compare the performance of these four models, the classification metrics are calculated on the test dataset for the reconstructed CREF at different thresholds in Figure 6. The result shows that when the CREF is small, the POD, CSI, and HSS scores are high for all models. With the increase of CREF to 40 dBZ, the POD, CSI, and HSS scores of the Attention U-Net model are decreased from about 0.9 to 0.3. However, the POD, CSI, and HSS values of the Attention U-Net model are still significantly superior to those of the U-Net model. Similarly, the FAR values are lower for the Attention U-Net and U-Net models at low radar echo thresholds. As the radar echo increases to about 35–40 dBZ, the FAR values increase to about 0.35, but the Attention U-Net model is still better than the U-Net model. In addition, the performance of the VIS+IR and the IR-only models are also compared to verify the added value of VIS/NIR bands. The results reveal that adding VIS/NIR bands can improve the model performance, especially for the U-Net model. When the radar echo increases to 30 dBZ, the POD, CSI, and HSS of the U-Net VIS_IR model are significantly better than those of the U-Net IR model. In general, through the comparison between the four models, it is found that the AU_VIS_IR model has the best classification scores, with the POD, CSI, and HSS being higher than 0.8 and the FAR value lower than 0.1 at a low radar echo threshold. As the radar echo threshold increases, the performance gradually becomes worse. However, the AU_VIS_IR model is still superior to the rest models. The AU model achieves better results than the U model in reconstructing the CREF and the VIS+IR model also exhibit better performance than the IR-only model. However, all four models show deficiencies in regions of strong radar reflectivity.

**Figure 7:**
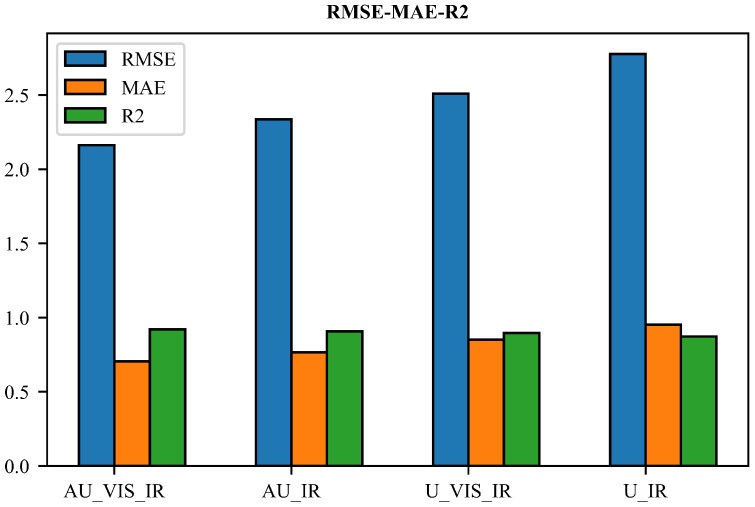
Regression metrics of the 4 models on the test dataset.

Figure 7 shows the results of RMSE, MAE, and R2 for the four models on the test dataset. The RMSE, MAE, and R2 of the AU_VIS_IR model are 2.1926 dBZ, 0.7107 dBZ, and 0.9176, respectively. The RMSE, MAE, and R2 of the AU_IR model are 2.3849 dBZ, 0.7764 dBZ, and 0.9027, respectively. The AU_VIS_IR model has better performance than the AU_IR model in terms of all statistics. The situation is similar for the U_VIS_IR model and the U_IR model. The RMSE, MAE, and R2 of the U_VIS_IR model are 2.5024 dBZ, 0.8457 dBZ, and 0.8954, respectively, which are superior to those of the U_IR model. This is probably because the VIS/NIR bands include additional information on cloud optical depth, including VIS/IR bands, which could better reflect the evolution of cloud and convective systems [28]. Overall, the AU_VIS_IR model shows the best performance among all models in terms of all statistics, which is consistent with the results mentioned above.

### 4.2. Case Study Analysis

**Figure 8:**
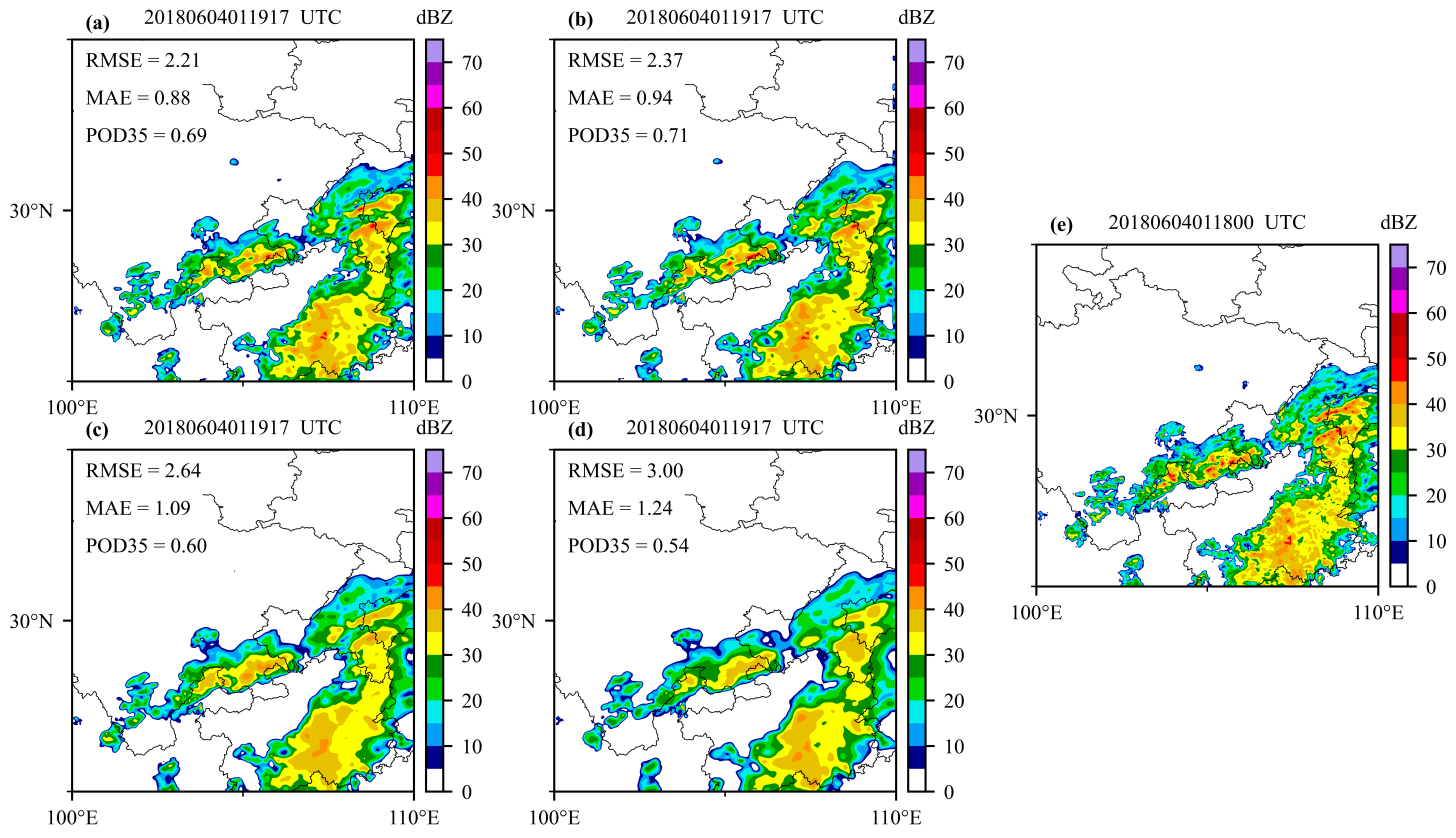
Reconstructed CREF based on satellite observation. (**a**) Attention U-Net VIS_IR model; (**b**) Attention U-Net IR model; (**c**) U-Net VIS_IR model; (**d**) U-Net IR model; (**e**) actual observed CREF.

In this section, a strong convective case is taken to display the reconstruction performance of the four models. Figure 8 shows a heavy precipitation process that occurred around 01:00 UTC on 4 June 2018, hitting the southeast of Sichuan province, and most areas of Chongqing and Guizhou province. This heavy rainfall process was characterized by long duration, short-term strong rainfall, and distinct local features.

It is shown that the reconstruction of CREF from four models matches well with the actual radar observations. The four models can basically reconstruct the distribution pattern of actual observation data and capture the center of the strong convective system. However, there are some missing details in the four models, especially in the U-Net model, which may be related to the low spatial resolution of the satellite in comparison to the radar. Moreover, the reconstructed CREF based on the DL technique still has some limitations, and the maximum value is smaller than the actual observation. Overall, the Attention U-Net model can capture more detailed information compared with the U-Net model and is closer to the actual observations.

### 4.3. Application

**Figure 9:**
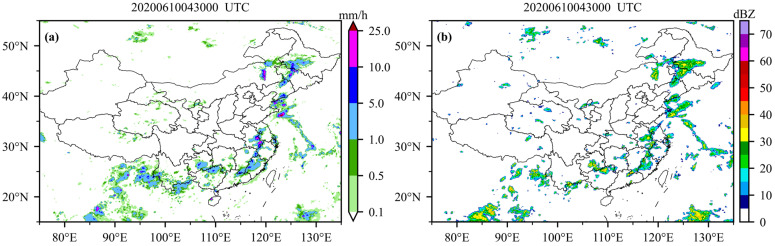
June 10, 2020, 04:30 UTC. (**a**) GPM precipitation distribution; (**b**) radar composite reflectivity based on the Attention U-Net VIS_IR model.

Based on the above analysis, the Attention U-Net VIS_IR model with the best reconstruction performance is selected to generate the nationwide CREF. Because the actual radar observations are limited to spatial coverage, there is a large radar data void over the ocean and most areas of western China. In order to validate the reconstructed radar CREF from satellite, especially in the region where there is no actual radar observation, the GPM precipitation dataset is selected as supplementary information to indicate the area of strong radar echoes. Figure 9 shows a precipitation event from the GPM dataset and the corresponding reconstructed CREF map based on the AU_VIS_IR model at 04:30 UTC on 10 June 2020.

Starting from June 2020, the western Pacific subtropical high has continued developing and enhancing. The lower troposphere over the northwest Pacific is dominated by the anomalous anticyclonic wind and thus the water vapor transportation over the west side of the subtropical high is significantly stronger. This stronger transportation provides an unusual amount of water vapor from the South China Sea and the western Pacific for this persistent precipitation process in the southern region of China. From 2 to 10 June 2020, the east of the southern regions of China received heavy and widespread precipitation. This precipitation process was characterized by a wide area with a long duration and heavy rainfall. The accumulated precipitation exceeds 600 mm in some local areas of Guangdong and Guangxi Province. As shown in Figure 9a, strong rainfall occurred across northern Guangxi, the southern region of Guizhou, western Fujian, and the middle-lower reaches of the Yangtze River region, with the rain rate exceeding 20 mm/h in Anhui, Inner Mongolia, and Jilin. Figure 9b shows the reconstructed CREF map from the AU_VIS_IR model with AGRI observations at the corresponding time.

Overall, the distribution of reconstructed CREF is quite consistent with the pattern of the precipitation, with the maximum CREF up to approximately 45 dBZ. The region of strong reconstructed radar echoes is consistent with the short-term heavy precipitation areas, such as the southern Anhui, north China, and the western Pacific, where the reconstructed CREF reaches above 30 dBZ and the rain rate is over 10 mm/h. Therefore, the CREF retrieved from satellite observations with the DL model can be used as a good indicator of heavy precipitation and provide relatively reliable supplementary information in the regions where the coverage of radar is incomplete. However, the model also has missed some precipitation areas. For example, there is a strong precipitation center that occurs over Hainan Island, but it is not shown on the reconstructed radar map. Moreover, there are also some regions with strong radar echoes but no precipitation. In the future, adding NWP model data can be considered in the training process to improve the model’s ability to capture severe weather.

## 5. Summary and Conclusions

This study aims to develop a retrieval algorithm for radar reflectivity from observations of the Chinese new-generation geostationary meteorological satellite FY-4A, to take advantage of the large coverage of the GEO satellite, and make up for the deficiencies of radar observations. The U-Net and Attention U-Net models are utilized to build the VIS+IR model and the IR-only model, respectively. This study also performs a comparative analysis of the above four models, and the results show the DL models could well reconstruct the radar reflectivity from satellite observations, but they also show deficiencies in the regions with strong radar echoes. The Attention U-Net model is superior to the other three models in terms of all classification scores and statistics. Moreover, adding the VIS/NIR bands as input in the model can improve the performance compared with using IR bands only.

Besides the calculation of statistics, the reconstruction ability is also validated. The examination is carried out using actual radar observations over the area within the radar coverage. The comparison results reveal that the DL models could rebuild the shape and location of actual radar echoes, while the intensities are somewhat underestimated. When compared with the U-Net model, the Attention U-Net model can capture more detailed information about the radar echoes and produce closer results to the actual radar observations. Therefore, the Attention U-Net model is selected to generate a radar CREF map with wide coverage to analyze a typical widespread precipitation process. The GPM precipitation dataset is utilized for independent validation, especially for regions without radar observations. The reconstructed CREF from FY-4A AGRI data is consistent with the pattern of the strong precipitation signals and could be used as a relatively reliable indicator of strong precipitation to compensate for the lack of radar observations. However, the GEO satellite IR images mainly observe the cloud top information, which is different from the principle and method of radar detection. Only using satellite observations as model input data may cause large uncertainties under some circumstances, such as extreme cases with strong radar echoes. Future work will be focused on improving radar reflectivity retrieval under situations with strong radar echoes, for example, through building a more representative training dataset. In addition, since the NWP model data can provide further information on clouds and strong convections, adding cloud hydrometers from the NWP model is another approach to further improve the model performance. Moreover, how to blend satellite reconstructed radar with actual radar observations to form a unified radar reflectivity field with larger coverage and higher frequency for better applications also needs to be studied in the future.

## Figures and Tables

**Table 1:** Satellite characteristic parameters and physical significance of model inputs.

NO	Input Factor	Physical Meaning
1	0.65 μm	Cloud optical thickness, strong convective clouds phase
2	1.61 μm	Ice-phase clouds, cloud effective particle radius
3	2.225 μm	Cloud phase, aerosol, vegetation
4	3.725 μm	Surface
5	10.8 μm	Cloud top temperature estimation
6	12.0 μm	Cloud top temperature estimation
7	10.8–6.2 μm	Cloud top height relative to the convective layer
8	12.0 + 8.5 − 2 × 10.8 μm	Cloud top phase state
9	DEM	Regional topography
VIS + IR	Inputs: 1–9	
IR-only	Inputs: 4–9	

**Table 2:** Definition of classification index parameters.

		Observation	
		T	F
Estimation	T	TP	FP
	F	FN	TN

## Data Availability

The AGRI data are from http://satellite.nsmc.org.cn/PortalSite/Default.aspx, accessed on 1 October 2021, the GPM precipitation data are obtained from https://gpm.nasa.gov/data/directory accessed on 1 November 2021 and the radar composite reflectivity data were generously shared by China Radar Mosaic System V3.0 (CRMS3.0) and Chinese National Meteorological Information Center.

## References

[1] Xu X., Tang Q. (2021). Meteorological disaster frequency at prefecture-level city scale and induced losses in mainland China during 2011–2019. Nat. Hazards.

[2] Rigo T., Castillo S. (2021). Evolution of Radar and Lightning Variables in Convective Events in Barcelona and Surroundings for the Period 2006–2020. Adv. Environ. Eng. Res..

[3] Nastos P.T., Dalezios N.R., Faraslis I.N., Mitrakopoulos K., Blanta A., Spiliotopoulos M., Sakellariou S., Sidiropoulos P., Tarquis A.M. (2021). Risk management framework of environmental hazards and extremes in Mediterranean ecosystems. Nat. Hazards Earth Syst. Sci..

[4] Wang L., Li Y., Xu X., Li F. (2021). Characteristic Analysis of dual-polarization weather radar echoes of convective precipitation and snowfall in the Mount Everest Region. Atmosphere.

[5] Borga M., Marra F., Gabella M. (2022). Rainfall estimation by weather radar. Rainfall.

[6] Binetti M.S., Campanale C., Massarelli C., Uricchio V.F. (2022). The Use of Weather Radar Data: Possibilities, Challenges and Advanced Applications. Earth.

[7] Roberts R.D., Rutledge S. (2003). Nowcasting storm initiation and growth using GOES-8 and WSR-88D data. Weather Forecast..

[8] Zeng Z., Wang D., Chen Y. (2021). An investigation of convective features and ZR relationships for a local extreme precipitation event. Atmos. Res..

[9] Sokol Z. (2011). Assimilation of extrapolated radar reflectivity into a NWP model and its impact on a precipitation forecast at high resolution. Atmos. Res..

[10] Sokol Z., Zacharov P. (2012). Nowcasting of precipitation by an NWP model using assimilation of extrapolated radar reflectivity. Q. J. R. Meteorol. Soc..

[11] Meyers M.P., Steenburgh W.J. (2013). Mountain weather prediction: Phenomenological challenges and forecast methodology. Mountain Weather Research and Forecasting.

[12] AghaKouchak A., Farahmand A., Melton F., Teixeira J., Anderson M., Wardlow B.D., Hain C. (2015). Remote sensing of drought: Progress, challenges and opportunities. Rev. Geophys..

[13] Wang G., Wang K., Han W., Wang D., Qiu X. (2020). Typhoon Maria precipitation retrieval and evolution based on the infrared brightness temperature of the Feng-Yun 4A/advanced geosynchronous radiation imager. Adv. Meteorol..

[14] Mecikalski J.R., Bedka K.M. (2006). Forecasting convective initiation by monitoring the evolution of moving cumulus in daytime GOES imagery. Mon. Weather Rev..

[15] Vila D.A., Machado L.A.T., Laurent H., Velasco I. (2008). Forecast and Tracking the Evolution of Cloud Clusters (ForTraCC) using satellite infrared imagery: Methodology and validation. Weather Forecast..

[16] Ren J., Xu G., Zhang W., Leng L., Xiao Y., Wan R., Wang J. (2021). Evaluation and Improvement of FY-4A AGRI Quantitative Precipitation Estimation for Summer Precipitation over Complex Topography of Western China. Remote Sens..

[17] Yang J., Zhang Z., Wei C., Lu F., Guo Q. (2017). Introducing the new generation of Chinese geostationary weather satellites, Fengyun-4. Bull. Am. Meteorol. Soc..

[18] Rutledge S., Hilburn K., Clayton A., Fuchs B., Miller S. (2020). Evaluating Geostationary Lightning Mapper flash rates within intense convective storms. J. Geophys. Res. Atmos..

[19] Hilburn K.A., Ebert-Uphoff I., Miller S.D. (2021). Development and interpretation of a neural-network-based synthetic radar reflectivity estimator using GOES-R satellite observations. J. Appl. Meteorol. Climatol..

[20] Zhou K., Zheng Y., Li B., Dong W., Zhang X. (2019). Forecasting different types of convective weather: A deep learning approach. J. Meteorol. Res..

[21] Han L., Sun J., Zhang W. (2019). Convolutional neural network for convective storm nowcasting using 3-D Doppler weather radar data. IEEE Trans. Geosci. Remote Sens..

[22] Sadeghi M., Asanjan A.A., Faridzad M., Nguyen P., Hsu K., Sorooshian S., Braithwaite D. (2019). Persiann-cnn: Precipitation estimation from remotely sensed information using artificial neural networks–convolutional neural networks. J. Hydrometeorol..

[23] Tian L., Li X., Ye Y., Xie P., Li Y. (2019). A generative adversarial gated recurrent unit model for precipitation nowcasting. IEEE Geosci. Remote Sens. Lett..

[24] Rüttgers M., Lee S., Jeon S., You D. (2019). Prediction of a typhoon track using a generative adversarial network and satellite images. Sci. Rep..

[25] Fukami K., Fukagata K., Taira K. (2021). Machine-learning-based spatio-temporal super resolution reconstruction of turbulent flows. J. Fluid Mech..

[26] Wang P., Fan E., Wang P. (2021). Comparative analysis of image classification algorithms based on traditional machine learning and deep learning. Pattern Recognit. Lett..

[27] Duan M., Xia J., Yan Z., Han L., Zhang L., Xia H., Yu S. (2021). Reconstruction of the Radar Reflectivity of Convective Storms Based on Deep Learning and Himawari-8 Observations. Remote Sens..

[28] Sun F., Li B., Min M., Qin D. (2021). Deep Learning-Based Radar Composite Reflectivity Factor Estimations from Fengyun-4A Geostationary Satellite Observations. Remote Sens..

[29] Min C., Chen S., Gourley J.J., Chen H., Zhang A., Huang Y., Huang C. (2019). Coverage of China new generation weather radar network. Adv. Meteorol..

[30] Sharifi E., Steinacker R., Saghafian B. (2016). Assessment of GPM-IMERG and other precipitation products against gauge data under different topographic and climatic conditions in Iran: Preliminary results. Remote Sens..

[31] Wehbe Y., Temimi M., Adler R.F. (2020). Enhancing precipitation estimates through the fusion of weather radar, satellite retrievals, and surface parameters. Remote Sens..

[32] Hou A.Y., Kakar R.K., Neeck S., Azarbarzin A.A., Kummerow C.D., Kojima M., Oki R., Nakamura K., Iguchi T. (2014). The global precipitation measurement mission. Bull. Am. Meteorol. Soc..

[33] Tang S., Li R., He J., Wang H., Fan X., Yao S. (2020). Comparative evaluation of the GPM IMERG early, late, and final hourly precipitation products using the CMPA data over Sichuan Basin of China. Water.

[34] Wang X., Min M., Wang F., Guo J., Li B., Tang S. (2019). Intercomparisons of cloud mask products among Fengyun-4A, Himawari-8, and MODIS. IEEE Trans. Geosci. Remote Sens..

[35] Ronneberger O., Fischer P., Brox T. (2015). U-net: Convolutional networks for biomedical image segmentation. Proceedings of the International Conference on Medical Image Computing and Computer-Assisted Intervention.

[36] Gao Y., Guan J., Zhang F., Wang X., Long Z. (2022). Attention-Unet-Based Near-Real-Time Precipitation Estimation from Fengyun-4A Satellite Imageries. Remote Sens..

[37] Oktay O., Schlemper J., Folgoc L.L., Lee M., Heinrich M., Misawa K., Mori K., McDonagh S., Hammerla N.Y., Kainz B. (2018). Attention u-net: Learning where to look for the pancreas. arXiv.

[38] Xiong R., Yang Y., He D., Zheng K., Zheng S., Xing C., Zhang H., Lan Y., Wang L., Liu T. On layer normalization in the transformer architecture. Proceedings of the International Conference on Machine Learning (PMLR).

[39] Liu L., Jiang H., He P., Chen W., Liu X., Gao J., Han J. (2019). On the variance of the adaptive learning rate and beyond. arXiv.

